# Apple Seed Extract Post-Treatment Alters Selected IGF-Related and Extracellular Matrix-Associated Markers Following Tobacco Leaf Extract-Induced Histological Liver Injury

**DOI:** 10.3390/ijms27135851

**Published:** 2026-06-29

**Authors:** Min Jee Oh, Yong-Su Park, Ji-Yeon Mo, Eun Kyung Kang, Cheol Won Kang, Sang Hwan Kim

**Affiliations:** 1 General Graduate School of Animal Life Convergence Science, Hankyong National University, 327, Jungang-ro, Ansung 17579, Gyeonggi-do, Republic of Korea; wertey08@naver.com (M.J.O.); mjy010627@naver.com (J.-Y.M.); nice-luck@hanmail.net (E.K.K.); elbin0674@naver.com (C.W.K.); 2 School of Animal Life Convergence Science, Hankyong National University, 327, Jungang-ro, Ansung 17579, Gyeonggi-do, Republic of Korea; 3 Research Center for Endangered Species, National Institute of Ecology, 1210, Geumgang-ro, Maseo-myeon, Seocheon-gun 33657, Chungcheongnam-do, Republic of Korea; muskdeer@nie.re.kr; 4 Institute of Applied Humanimal Science, Hankyong National University, 327, Jungang-ro, Ansung 17579, Gyeonggi-do, Republic of Korea

**Keywords:** tobacco leaf extract, apple seed extract, liver histology, apoptosis, extracellular matrix, IGF-related markers

## Abstract

Tobacco leaf extract (TLE) exposure can induce liver injury-associated responses involving cell death, inflammatory signaling, and extracellular matrix (ECM)-related changes. This study examined whether apple seed extract (ASE) post-treatment changes apoptosis-, inflammation-, ECM-, and insulin-like growth factor (IGF)-related markers after TLE exposure. Primary mouse hepatocytes were exposed to TLE, ASE alone, or TLE followed by ASE, and mouse liver tissues were examined after TLE exposure with or without ASE post-treatment. TLE reduced hepatocyte viability in a concentration-dependent manner, with an IC_50_ of 4.4 mg/mL. Annexin V/propidium iodide analysis showed that early apoptosis increased from 2.20% in untreated cells to 5.50% after 2 mg/mL TLE and 85.65% after 4 mg/mL TLE. ASE alone at 40 µg/mL increased the early apoptotic fraction to 53.65%, indicating that this concentration was not biologically neutral under basal culture conditions. After TLE exposure followed by ASE post-treatment, the live-cell fraction remained high in T2HA and T4HA, whereas T5HA retained a high early apoptotic fraction. In mice, TLE exposure was accompanied by visible liver appearance changes and histological alterations. ASE post-treatment changed Alcian blue staining, gelatinase activity, TIMP-associated signals, and IGF-related signals. These findings indicate treatment-dependent changes in selected injury-associated markers rather than a consistently protective effect of ASE. The study does not assign these effects to a specific ASE constituent because compound-level chemical standardization was not performed.

## 1. Introduction

The liver is a major site for xenobiotic metabolism and is therefore vulnerable to toxicant-associated oxidative and inflammatory stress. Tobacco-derived toxicants have been linked to systemic injury responses, and clinical studies have reported associations between cigarette smoking and liver disease severity, including steatotic liver disease and fibrosis-related progression [1,2,3]. Experimental cigarette smoke exposure has also been reported to aggravate steatosis-to-steatohepatitis progression in high-fat-diet-fed mice through oxidative stress-related mechanisms [4]. Although clinical smoking exposure, cigarette smoke exposure, and experimental TLE exposure are not identical, these findings provide a rationale for investigating liver responses to tobacco-derived injury stimuli.

Liver injury involves overlapping cellular and tissue-level responses, including oxidative stress, inflammatory signaling, apoptosis, and matrix remodeling. Apoptosis contributes to tissue damage and is regulated by BCL-2 family proteins and caspase activation [5]. Tobacco-derived toxicants can induce apoptosis and necrosis to different extents depending on exposure intensity, chemical composition, and biological context [2,3,6]. These findings indicate that cell death profiles and inflammatory readouts are useful markers for evaluating toxicant-associated cellular injury, although they do not by themselves establish functional tissue recovery.

Tissue-level injury is also accompanied by changes in the extracellular matrix (ECM). ECM remodeling is closely linked to liver injury, repair, fibrosis-related progression, and fibrosis regression, and matrix metalloproteinases (MMPs) and tissue inhibitors of metalloproteinases (TIMPs) participate in ECM turnover during these processes [7,8,9,10]. MMP-2 and MMP-9 are frequently used as gelatinase-associated markers of ECM remodeling [7]. However, MMP/TIMP changes alone are not sufficient to determine collagen deposition, myofibroblast activation, or hepatic fibrosis progression. Therefore, the ECM-related interpretation in the present study was limited to ECM-associated marker changes.

Growth factor-related signaling is another component of the liver response to injury. The insulin-like growth factor (IGF) system is associated with hepatic metabolism, cell survival, and regeneration, and altered IGF-related signaling has been reported in liver disease [8,11,12]. However, changes in IGF-related proteins alone do not establish activation of a complete signaling pathway. In the present study, IGF-related proteins were therefore evaluated as selected survival-associated markers rather than as standalone evidence of pathway activation.

Natural products remain of interest in liver injury research because crude extracts often contain multiple bioactive constituents. Apple seeds contain phenolic compounds, cyanogenic glycosides, lipids, and other phytochemical constituents, including amygdalin and phloridzin (phlorizin)-related compounds [10,13,14]. Previous studies using purified or defined compounds have reported that amygdalin and phloridzin can affect inflammation-, oxidative stress-, and apoptosis-related pathways in experimental injury models [15,16]. In addition, previous ASE-based studies reported changes in liver injury-, tissue remodeling-, IGF-related, and apoptosis-associated readouts, providing a basis for further evaluating ASE as a crude extract in injury-related models [17,18]. However, these findings cannot be directly assigned to a single apple seed-derived compound unless the extract is chemically characterized. In this study, ASE was used as a whole extract prepared on a dried extract equivalent basis, and its effects were interpreted at the crude-extract level rather than at the level of a single constituent.

Pre-treatment and co-treatment designs are commonly used in experimental studies evaluating natural products in liver injury models. A post-treatment design is useful for examining tissue responses after toxic exposure has already occurred. In the present study, we examined whether ASE post-treatment changes histological injury patterns and selected apoptosis-, inflammation-, ECM-, and IGF-related markers after TLE exposure. The study was designed to evaluate treatment-associated changes in these readouts, not to assign the observed effects to a single ASE-derived compound, prove activation of a complete signaling pathway, or determine hepatoprotective efficacy.

## 2. Results

### 2.1. Tobacco Leaf Extract Induces Concentration-Dependent Cytotoxicity and Cell Death-Associated Changes in Hepatocytes

**Figure 1:**
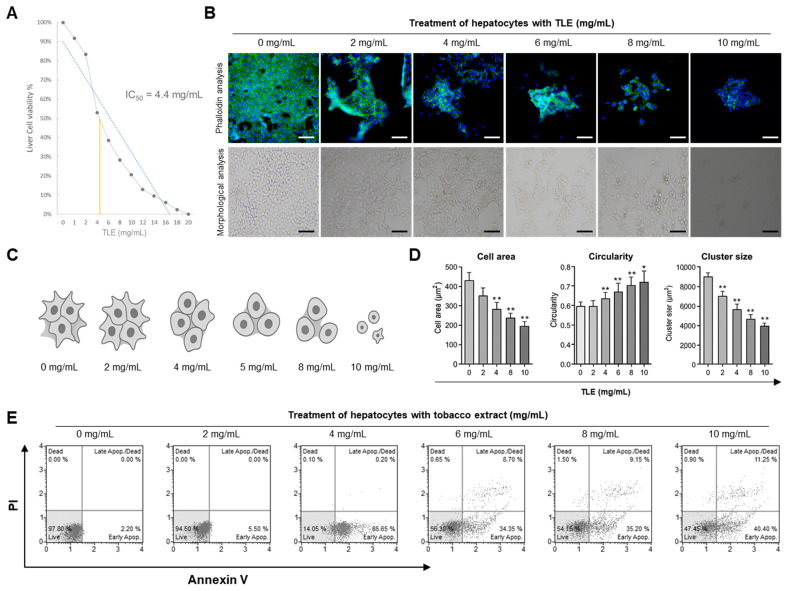
Tobacco leaf extract (TLE) induces concentration-dependent cytotoxicity and Annexin V/PI-defined cell death in hepatocytes. (**A**) Concentration–response curve of hepatocyte viability after 48 h exposure to TLE; the IC_50_ was 4.4 mg/mL. (**B**) Representative phalloidin-stained images (actin cytoskeleton) and corresponding bright-field images after 48 h TLE treatment (0–10 mg/mL). Images were acquired at 200× magnification. Scale bar, 100 µm. (**C**) Schematic illustration summarizing TLE-associated morphological changes from spread/clustered cells to rounded and dispersed cells. (**D**) Quantification of cell morphology showing decreased cell area, increased circularity, and reduced cluster size with increasing TLE concentration. (**E**) Representative Annexin V/propidium iodide (PI) flow cytometry plots showing changes in cell death profiles following TLE treatment. Quantitative data are presented as mean ± SEM (*n* = 3 independent experiments, each performed in technical triplicate). As shown in the figure, * *p* < 0.05 and ** *p* < 0.01 versus the untreated control group.

When TLE was applied to hepatocytes for 48 h, cell viability decreased with increasing TLE concentration, and concentration–response curve analysis estimated an IC_50_ of 4.4 mg/mL (Figure 1A). Phalloidin staining and morphological observations showed reduced cell density and disruption of clustered cell morphology after TLE exposure. These changes were accompanied by loss of actin cytoskeletal continuity and increased cell rounding (Figure 1B,C). Morphometric analysis showed reduced cell area, increased circularity, and decreased cluster size following TLE treatment (Figure 1D).

Annexin V/PI flow cytometry showed concentration-related changes in cell death profiles after TLE treatment (Figure 1E). In the control group (0 mg/mL), viable cells accounted for 97.80%, and early apoptotic cells accounted for 2.20%. At 2 mg/mL TLE, the early apoptotic fraction increased to 5.50%. At 4 mg/mL TLE, the viable fraction decreased to 14.05%, whereas the early apoptotic fraction increased to 85.65%. At 6–10 mg/mL TLE, the late apoptotic/PI-positive fraction increased, and the viable fraction further decreased (Figure 1E).

### 2.2. Apple Seed Extract Changes Apoptosis- and Inflammation-Associated Readouts in Hepatocytes

**Figure 2:**
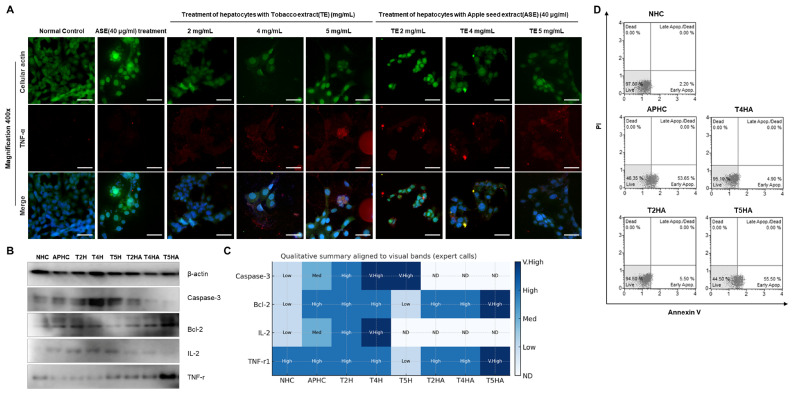
Apple seed extract (ASE) changes inflammatory and apoptotic readouts in hepatocytes. (**A**) Immunofluorescence images of cellular actin (phalloidin), TNF-α, and merged signals across treatment groups. Hepatocytes were treated with TLE alone (2, 4, or 5 mg/mL; T2H, T4H, T5H), ASE alone (40 µg/mL), or TLE followed by ASE (40 µg/mL; T2HA, T4HA, T5HA) as indicated. Scale bar, 100 µm. (**B**) Representative immunoblot images of caspase-3, BCL-2, IL-2, and TNF-R1 with β-actin as a loading control. (**C**) Qualitative summary map aligned to the immunoblot band patterns shown in (**B**), indicating relative signal changes across groups. (**D**) Representative Annexin V/PI flow cytometry plots comparing apoptotic distributions among NHC, APHC, and TLE followed by ASE groups. Quantitative values, where applicable, are presented as mean ± SEM (*n* = 3 independent experiments, each performed in technical triplicate).

The ASE-only group (APHC; 40 µg/mL) showed a marked change in Annexin V/PI distribution compared with the NHC group. The early apoptotic fraction increased from 2.20% in NHC to 53.65% in APHC, whereas the live-cell fraction decreased from 97.80% to 46.35% (Figure 2D). Immunofluorescence images also showed differences in cytoskeletal organization and TNF-α signal distribution between NHC and APHC (Figure 2A). These results indicate that ASE at 40 µg/mL changed hepatocyte survival and cellular morphology under basal culture conditions. This ASE-only response was therefore considered when interpreting the post-treatment groups.

Under TLE-only conditions, cell rounding and loss of cluster integrity were observed in a concentration-related manner (Figure 1B–D), and TNF-α immunofluorescence signal appeared more prominent than that in NHC (Figure 2A). Immunoblotting showed changes in apoptosis- and inflammation-associated proteins, including caspase-3, BCL-2, IL-2, and TNF-R1 (Figure 2B). These findings show that TLE exposure was accompanied by changes in cellular morphology and molecular readouts related to cell death and inflammation.

In groups treated with ASE after TLE exposure (T2HA, T4HA, and T5HA), TNF-α signal distribution, cell morphology, and immunoblot patterns differed among treatment groups (Figure 2A,B). Annexin V/PI flow cytometry showed that the live-cell fraction remained high in T2HA and T4HA groups (T2HA: live, 94.50%; early apoptosis, 5.50%; T4HA: live, 95.10%; early apoptosis, 4.90%). In contrast, the T5HA group showed 55.50% early apoptotic cells and 44.50% live cells, a pattern similar to that observed in APHC (Figure 2D). These results indicate that ASE post-treatment was not consistently associated with reduced apoptosis-associated profiles across all TLE exposure levels.

### 2.3. Apple Seed Extract Post-Treatment Is Associated with Changes in Gross Liver Appearance and Histological Patterns in TLE-Exposed Mice

**Figure 3:**
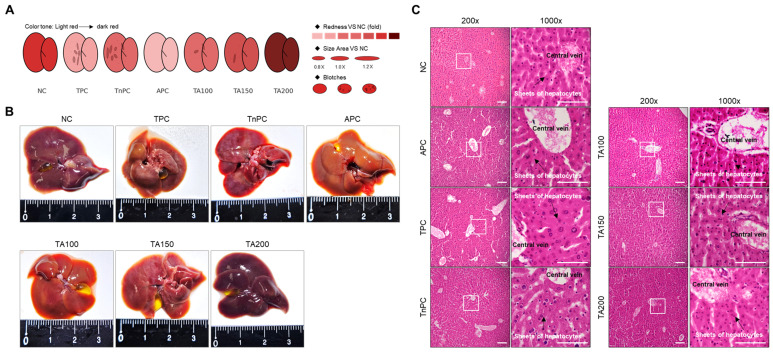
Apple seed extract (ASE) post-treatment is associated with changes in gross liver appearance and histological patterns in tobacco leaf extract (TLE)-exposed mice. (**A**) Schematic overview summarizing group-wise differences in gross liver appearance, including relative color tone (redness), apparent surface area, and the presence of surface blotches. These gross features were evaluated descriptively and were not used as quantitative indicators of liver injury. (**B**) Representative images of excised livers from each group. (**C**) Representative hematoxylin and eosin (H&E)-stained liver sections shown at 200× and 1000× magnifications. Compared with NC, TLE-exposed groups display histological alterations characterized by cytoplasmic vacuolation, inflammatory foci-like aggregates, and hepatocyte swelling with ballooning-like morphology. ASE-treated groups show group-dependent differences in these patterns. H&E sections were prepared at 10 µm thickness. Scale bars, 100 µm (200×) and 50 µm (1000×). NC: Non-treatment control; TPC: tobacco leaf extract (TLE; 5 mg per mouse/day)-treated control; TnPC: TLE-withdrawal group after 7 days of TLE administration; APC: apple seed extract (ASE)-only control; TA100: TLE + ASE 100 mg/kg post-treatment; TA150: TLE + ASE 150 mg/kg post-treatment; TA200: TLE + ASE 200 mg/kg post-treatment.

Gross examination showed that the normal control group (NC) exhibited a relatively uniform color tone and smooth surface morphology (Figure 3A,B). In contrast, the group treated with TLE for 14 days (TPC) showed a paler coloration and more irregular surface compared with the NC group, and the schematic summary also indicated the presence of surface blotches (Figure 3A,B). Similar gross changes were observed in the group switched to saline after seven days of TLE treatment (TnPC), indicating that gross morphological differences remained evident after TLE withdrawal (Figure 3A,B). The ASE-only group (APC) also showed gross features that differed from those of the normal controls (Figure 3A,B).

The post-treatment groups that received ASE after TLE exposure (TA100, TA150, and TA200) showed gross morphological patterns that differed from those observed in TPC and TnPC groups (Figure 3A,B). In the schematic summary, the treatment groups showed relatively increased apparent redness and fewer apparent surface blotches (Figure 3A), and the representative images showed differences in color tone and surface appearance among groups (Figure 3B). These gross findings were evaluated descriptively and were not used as quantitative indicators of liver injury.

Histological analysis showed that the NC group maintained a relatively uniform parenchymal architecture, whereas the TPC and TnPC groups displayed cytoplasmic vacuolation, inflammatory foci-like aggregates, and hepatocyte swelling with ballooning-like morphology (Figure 3C). The ASE-only group (APC) also showed histological features that differed from those of NC (Figure 3C). The TLE plus ASE post-treatment groups (TA100–TA200) showed group-specific differences in these histological patterns (Figure 3C). These observations show that TLE exposure and ASE post-treatment were accompanied by visible changes in liver appearance and tissue architecture, without establishing functional recovery.

### 2.4. ASE Post-Treatment Is Accompanied by Changes in ECM-Associated Staining and Selected Apoptosis-, Inflammation-, and IGF-Related Markers After TLE Exposure

Because gross and histological changes are described in Figure 3, the following analyses focused on ECM-associated staining and selected molecular markers in liver tissues. These tissue-level analyses were used to compare marker distribution and signal patterns among NC, TPC, TnPC, APC, and ASE post-treatment groups.

**Figure 4:**
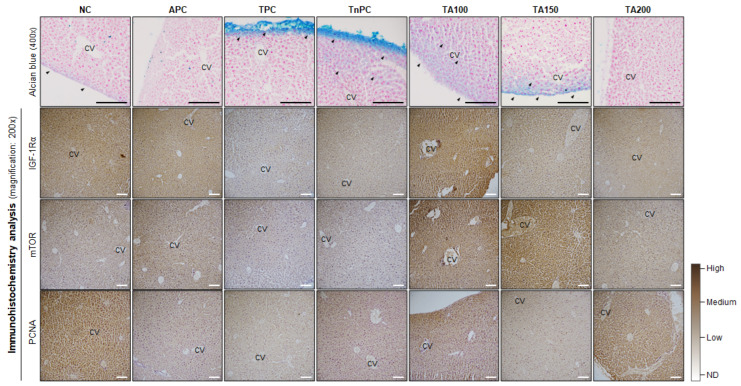
Apple seed extract (ASE) post-treatment is associated with Alcian blue staining patterns and survival/proliferation-associated protein signals in liver tissues following tobacco leaf extract (TLE) exposure. Representative images of Alcian blue staining (400×) and immunohistochemistry for IGF-IRα, mTOR, and PCNA (200×) in mouse liver sections from each group. Scale bars: 100 µm. NC: Non-treatment control; TPC: tobacco leaf extract (TLE; 5 mg per mouse/day)-treated control; TnPC: TLE-withdrawal group after 7 days of TLE administration; APC: apple seed extract (ASE)-only control; TA100: TLE + ASE 100 mg/kg post-treatment; TA150: TLE + ASE 150 mg/kg post-treatment; TA200: TLE + ASE 200 mg/kg post-treatment. CV: Central vein. The black arrow indicates the representative reaction zone of Alcian blue.

Alcian blue staining showed group-specific differences in staining distribution (Figure 4). In TPC and TnPC groups, blue-stained regions appeared more extensive than those in NC, and the ASE-only group (APC) also showed a staining pattern that differed from NC. In TA100–TA200 groups, Alcian blue staining patterns differed from those observed in TPC and TnPC groups. These findings showed descriptive tissue-level differences in ECM-associated staining. Because collagen deposition staining was not performed, these results should not be interpreted as direct evidence of altered hepatic fibrosis progression.

Immunohistochemistry for IGF-IRα, mTOR, and PCNA also showed group-specific signal patterns in liver tissue (Figure 4). In TPC and TnPC groups, the distribution of these signals differed from NC, and APC also showed a pattern distinct from NC. In TA100–TA200 groups, IGF-IRα, mTOR, and PCNA signal distributions varied among ASE post-treatment groups. These results showed group-specific changes in survival- and proliferation-associated protein signal patterns.

**Figure 5:**
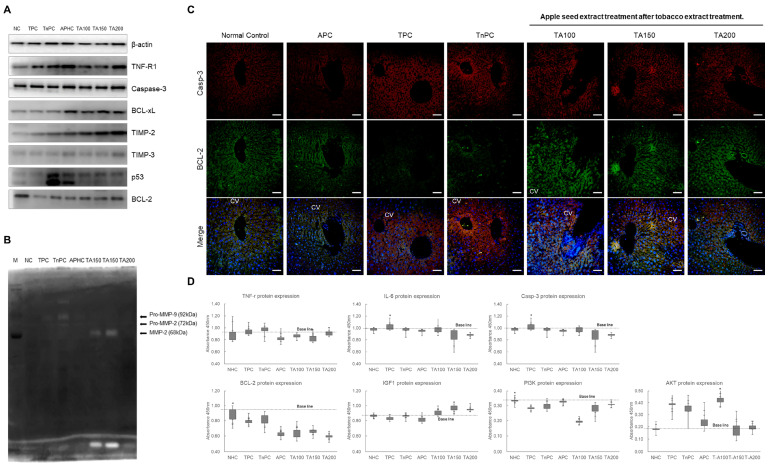
ASE post-treatment is associated with apoptosis-, inflammation-, and ECM-related protein readouts in liver tissues after TLE exposure. (**A**) Representative immunoblot images of TNF-R1, caspase-3, BCL-xL, TIMP-2, TIMP-3, p53, and BCL-2 with β-actin as a loading control. (**B**) Representative zymography showing gelatinase activity bands corresponding to pro-MMP-9, pro-MMP-2, and MMP-2. (**C**) Representative immunofluorescence images of caspase-3 (red) and BCL-2 (green) with nuclear counterstaining (DAPI, blue) across groups. Scale bars: 50 µm. (**D**) Relative absorbance signals for TNF-R1, IL-6, caspase-3, BCL-2, IGF-1, PI3K, and AKT measured using an antibody-based colorimetric assay. Statistical annotations are shown in the corresponding plots; * *p* < 0.05 was considered significant. These signals were used for within-assay comparison only and do not represent absolute protein concentrations. NC: Non-treatment control; TPC: tobacco leaf extract (TLE; 5 mg per mouse/day)-treated control; TnPC: TLE-withdrawal group after 7 days of TLE administration; APC: apple seed extract (ASE)-only control; TA100: TLE + ASE 100 mg/kg post-treatment; TA150: TLE + ASE 150 mg/kg post-treatment; TA200: TLE + ASE 200 mg/kg post-treatment. CV: Central vein.

Protein-level analyses showed group-specific changes in inflammation-, apoptosis-, and ECM-associated readouts (Figure 5). Western blot analysis showed changes in TNF-R1, caspase-3, p53, BCL-xL, TIMP-2, TIMP-3, and BCL-2 signals among treatment groups (Figure 5A). Gelatin zymography showed differences in bands corresponding to pro-MMP-9, pro-MMP-2, and MMP-2 (Figure 5B). Immunofluorescence analysis also showed group-specific caspase-3 and BCL-2 signal patterns in liver tissues (Figure 5C).

The antibody-based colorimetric assay showed group differences in relative absorbance signals for TNF-R1, IL-6, caspase-3, BCL-2, IGF-1, PI3K, and AKT (Figure 5D). IL-6 and caspase-3 showed the highest mean values in TPC, with significance indicated in the figure annotation (*p* < 0.05). BCL-2 and PI3K showed the highest mean values in NC, with significance indicated in the figure annotation (*p* < 0.05). IGF-1 showed the highest mean value in TA150, and AKT showed the highest mean value in TA100, with significance indicated in the figure annotation (*p* < 0.05). No significance marking was indicated for TNF-R1 in the figure; therefore, no between-group significance was reported for this marker in this panel. These data are presented as relative absorbance signals rather than absolute protein concentrations, and the group-specific patterns do not establish activation of a linear IGF-PI3K-AKT pathway.

## 3. Discussion

TLE exposure produced cellular and tissue-level changes involving reduced hepatocyte viability, altered Annexin V/PI profiles, cytoskeletal disruption, and histological liver alterations. These findings are consistent with previous reports showing that tobacco-derived toxicants and cigarette smoke exposure can promote oxidative stress, inflammatory signaling, and cell death-associated processes depending on exposure intensity and biological context [2,3,4,5,6,19]. In the present study, ASE post-treatment changed several apoptosis-, inflammation-, ECM-, and IGF-related markers after TLE exposure, but these changes did not follow a single uniform pattern across all experimental groups. Therefore, the present results should be interpreted as changes in selected histological and molecular readouts after TLE exposure, rather than as evidence of consistent or functional hepatoprotection by ASE.

In hepatocytes, TLE reduced viability, disrupted actin organization, reduced clustered cell morphology, and increased Annexin V/PI-defined cell death. These responses are compatible with toxicant-associated cellular injury, in which structural disruption and apoptotic signaling occur together [5,6,19]. Changes in caspase-3 and BCL-2 further support the involvement of apoptosis-related pathways because mitochondrial apoptosis is regulated by BCL-2 family proteins and downstream caspase activation [5,19]. Inflammatory signaling may also contribute to this injury response, as TNF-related pathways are known to interact with oxidative stress, JNK/IKK signaling, and cell death mechanisms during liver injury [20]. In the present study, changes in IL-2, TNF-R1, and TNF-α-associated signals indicate that inflammatory readouts were altered together with apoptosis-related markers after TLE exposure.

The tissue findings also support the presence of histological liver alterations after TLE exposure. TPC and TnPC groups showed cytoplasmic vacuolation, inflammatory foci-like aggregates, and ballooning-like hepatocyte morphology. Ballooning degeneration and inflammatory foci are commonly used histological features in liver injury assessment, particularly in scoring systems for steatohepatitis and related hepatic injury patterns [16]. However, serum biochemical markers of liver injury were not measured in this study. Therefore, the in vivo data should be interpreted as histological and tissue-level molecular findings, not as direct evidence of functional recovery of liver injury.

The ASE-only result is a key cautionary finding. ASE at 40 µg/mL increased the early apoptotic fraction in hepatocytes, indicating that this concentration was not neutral under basal culture conditions. This finding limits a simple protective interpretation of ASE and suggests that the crude extract may have treatment-context-dependent biological activity. In the post-treatment groups, T2HA and T4HA maintained high live-cell fractions, whereas T5HA showed an apoptotic profile similar to the ASE-only group. Thus, the cellular response to ASE post-treatment appeared to vary according to the preceding TLE concentration. However, this interpretation remains limited because no ASE dose–response experiment was performed and the extract was not chemically standardized. Future studies should define the cytotoxicity window of standardized ASE preparations before assigning hepatoprotective efficacy.

ECM-related markers also changed among treatment groups. ECM turnover is a central component of liver injury, repair, and fibrotic remodeling, and MMPs participate in matrix degradation, tissue remodeling, and inflammatory tissue responses [7,10,21,22]. MMP-2 and MMP-9 are frequently used as gelatinase-associated markers of ECM remodeling in liver injury and fibrosis-related contexts [7,21]. In the present study, gelatin zymography showed group differences in pro-MMP-9, pro-MMP-2, and MMP-2 bands, while TIMP-2 and TIMP-3 signals also differed among groups. These findings indicate that TLE exposure and ASE post-treatment changed ECM-associated readouts. However, the present data are not sufficient to define a transition from an MMP-9-dominant injury-clearance phase to an MMP-2-dominant reconstruction phase, nor do they establish altered hepatic fibrosis progression. Additional quantitative analyses, including densitometric validation, Sirius red or Masson’s trichrome staining, collagen quantification, and fibrosis-related markers such as α-SMA and TGF-β, would be needed to define the biological meaning of these ECM-related changes.

TIMP-2 and TIMP-3 also showed group-specific signal patterns, suggesting that MMP-associated changes occurred together with changes in endogenous MMP inhibitors. This is relevant because the balance between MMPs and TIMPs is an important determinant of ECM turnover during liver injury and fibrotic remodeling [10,21,22]. However, these findings should not be interpreted as proof of regulated ECM reconstruction without additional functional or histochemical evidence. The IGF-related results also require caution. The IGF system is associated with hepatic metabolism, survival signaling, and regenerative responses, and altered IGF signaling has been reported in liver disease [11,12,23]. In addition, mTOR signaling is broadly involved in cellular growth, metabolism, and stress-related adaptation [24]. Nevertheless, the present data did not show a single coordinated dose-related pattern across IGF-1, PI3K, AKT, and mTOR. IGF-1, AKT, and PI3K showed their highest values in different treatment groups. Therefore, the results indicate changes in IGF-related protein signals, but they do not establish activation of a linear IGF-PI3K-AKT-mTOR pathway by ASE.

The interpretation of ASE effects is also limited by the absence of chemical standardization. Apple seeds contain several bioactive constituents, including cyanogenic glycosides, phenolic compounds, and lipid-related components, and previous studies have reported amygdalin in apple seeds, apple-derived products, and apple seed meal [13,14,25,26]. Amygdalin has been reported to affect inflammation- and stress-associated pathways in an acute liver injury model, while phloridzin has been associated with changes in oxidative stress, inflammation, and apoptosis-related markers in hepatotoxicity models [15,16]. In addition, previous ASE-based studies reported changes in liver injury-, tissue remodeling-, IGF-related, and apoptosis-associated readouts, supporting the rationale for evaluating ASE at the crude-extract level [17,18]. However, the ASE used in the present study was not analyzed by HPLC, LC-MS, or compound-specific quantification. Therefore, the observed responses cannot be assigned to amygdalin, phloridzin, or any single apple seed-derived compound. The findings should be interpreted at the level of crude ASE treatment.

Several limitations should be considered when interpreting these findings. First, serum biochemical markers of liver injury were not measured, so the study cannot determine whether the histological and molecular changes correspond to functional improvement in liver injury. Second, although five mice were used per group, no a priori power calculation was performed. Third, ASE was not chemically standardized, and only one ASE concentration was used in the in vitro post-treatment experiments. Fourth, no reference hepatoprotective compound was included, so the relative efficacy of ASE could not be compared with a positive-control drug such as silybin. Fifth, collagen deposition staining and fibrosis-related markers were not assessed; therefore, the ECM findings should be interpreted as ECM-associated marker changes rather than evidence of altered hepatic fibrosis progression. These limitations restrict causal and translational interpretation. Nevertheless, the study shows that ASE post-treatment changed selected apoptosis-, inflammation-, ECM-, and IGF-related markers after TLE exposure. Future studies should combine liver enzyme analysis, standardized ASE preparations, ASE dose–response testing, positive-control treatment, fibrosis marker assessment, and direct pathway validation to determine whether ASE has reproducible effects on liver injury-related outcomes.

## 4. Materials and Methods

### 4.1. Ethics Statement and Animals

All animal procedures were approved by the Institutional Animal Care and Use Committee of Hankyong National University (approval no. HK-2024-2). Male Institute of Cancer Research (ICR) mice aged 12 weeks were used in this study. The animals were housed under controlled conditions (20 ± 1 °C, 50 ± 5% humidity, 12 h light/12 h dark cycle) with free access to food and water and were acclimatized for two weeks before the experiments.

### 4.2. Preparation of Tobacco Leaf Extract and Apple Seed Extract

TLE and ASE were prepared from dried tobacco leaf powder (80 g) and ground apple seed powder (50 g), respectively. Each material was extracted with 80% ethanol (32221; Sigma-Aldrich, St. Louis, MO, USA) at a 1:10 (*w*/*v*) ratio for 24 h at room temperature with continuous stirring. The extracts were concentrated under reduced pressure in a rotary evaporator (Büchi R-300; Büchi Labortechnik AG, Flawil, Switzerland), reconstituted in sterile 0.9% saline (S-4396; Sigma-Aldrich), and passed through a 0.22 µm syringe filter (SLGP033RS; Millipore, Burlington, MA, USA). Stock concentrations were calculated on a dried extract equivalent basis and adjusted to 2000 mg/mL for TLE and 15,800 mg/mL for ASE. Compound-level phytochemical standardization of the extracts, including HPLC- or LC-MS-based quantification, was not performed in this study. Stock solutions were stored at 4 °C and diluted in sterile saline for animal experiments or in culture medium for cell experiments immediately before use.

### 4.3. Isolation and Culture of Primary Mouse Hepatocytes

Primary mouse hepatocytes were aseptically isolated from liver tissues obtained from 12-week-old male ICR mice. Single-cell suspensions were prepared by enzymatic dissociation, filtration, and centrifugation according to the laboratory protocol used for this study. The cells were cultured in Dulbecco’s modified Eagle’s medium (DMEM; 11995065, Gibco, Thermo Fisher Scientific, Waltham, MA, USA) supplemented with 10% fetal bovine serum (FBS; 16000044, Gibco) and 1% antibiotic-antimycotic solution (15240062, Gibco) at 37 °C in a humidified incubator containing 5% CO_2_. All in vitro treatments were initiated when cells reached approximately 70% confluence. The isolated adherent cell population was used as the primary hepatocyte culture model for the in vitro experiments.

### 4.4. In Vitro Treatment Design

For concentration–response analysis, hepatocytes were exposed to TLE at 0, 2, 4, 6, 8, or 10 mg/mL for 48 h. Subsequent experiments were conducted using the following experimental groups: normal hepatocyte control, designated as NHC; TLE 2 mg/mL (T2H); TLE 4 mg/mL (T4H); TLE 5 mg/mL (T5H); ASE-only control (APHC); and TLE followed by ASE post-treatment (T2HA, T4HA, and T5HA). The APHC group received ASE alone at 40 µg/mL. For post-treatment experiments, cells were first exposed to TLE at 2, 4, or 5 mg/mL for 24 h, after which the medium was replaced and ASE was added at 40 µg/mL for an additional 24 h. A single ASE concentration was used for the in vitro post-treatment experiments; therefore, these experiments were not designed to determine ASE dose–response relationships. Quantitative cell-based assays were performed in at least three independent experiments, each conducted in technical triplicate.

### 4.5. Cell Viability Assay and IC_50_ Calculation

Cell viability was assessed using Cell Counting Kit-8 (CCK-8; Biomax, Guri city, Republic of Korea). Hepatocytes were seeded into 96-well plates and treated as indicated. After treatment, 10 µL of CCK-8 solution was added to each well and incubated for 1 h at 37 °C. Absorbance was measured at 450 nm using a microplate reader (Epoch Microplate Spectrophotometer; Agilent BioTek, Agilent Technologies, Santa Clara, CA, USA). Cell viability was expressed as a percentage of the untreated control. The half-maximal inhibitory concentration (IC_50_) of TLE was calculated from the concentration–response curve using four-parameter logistic nonlinear regression.

### 4.6. Phalloidin Staining

To assess the cytoskeletal changes, the treated cells were washed with phosphate-buffered saline (PBS), fixed in 4% paraformaldehyde for 15 min at room temperature, and permeabilized with 0.1% Triton X-100 for 5 min. The cells were then stained with phalloidin reagent (Abcam, Cambridge, UK) according to the manufacturer’s instructions. Fluorescence images were acquired using an ECLIPSE Ti2-U inverted fluorescence microscope (Nikon, Tokyo, Japan) under identical exposure conditions.

### 4.7. Annexin V/Propidium Iodide Flow Cytometry

Cell death was analyzed using Annexin V-FITC/propidium iodide (PI) staining. After treatment, the cells were harvested, washed twice with ice-cold PBS, and resuspended in binding buffer. Annexin V-FITC and PI were applied using the EzWay Annexin V-FITC Apoptosis Detection Kit (K29100; LABIS KOMA, Seoul, Republic of Korea) according to the manufacturer’s instructions. After incubation for 15 min at room temperature in the dark, samples were analyzed using a Guava Muse Cell Analyzer (Cytek Biosciences, Fremont, CA, USA). The proportions of viable, early apoptotic, late apoptotic, and necrotic cells were recorded.

### 4.8. Animal Experimental Design

A total of 35 male ICR mice were randomly assigned to seven groups (*n* = 5 per group): NC, TPC, TnPC, APC, TA100, TA150, and TA200. The NC group received 0.9% saline intraperitoneally once daily for 14 days. The TPC group received 5 mg of TLE per mouse via oral gavage once daily for 14 days. The TnPC group received 5 mg of TLE per mouse via oral gavage for seven days, followed by intraperitoneal saline for an additional seven days. The APC group received 200 mg/kg ASE intraperitoneally once daily for 14 days. The TA100, TA150, and TA200 groups received TLE (5 mg per mouse) via oral gavage for seven days, followed by ASE (100, 150, or 200 mg/kg) intraperitoneally once daily for seven days. TLE was administered as a fixed dose of 5 mg per mouse per day and was not adjusted according to individual body weight. Intraperitoneal injections were administered bilaterally in the lower abdomen at a volume of 50 µL per side. No positive-control hepatoprotective compound, such as silybin, was included, and no a priori power calculation was performed for the animal experiment.

### 4.9. Tissue Collection and Fixation

At the end of the treatment period, mice were euthanized by cervical dislocation. Blood samples were not collected in this experimental design; therefore, serum biochemical markers of liver injury, including alanine aminotransferase, aspartate aminotransferase, alkaline phosphatase, bilirubin, and albumin, were not measured. The livers were immediately excised, rinsed in 1× PBS, and photographed for gross examination. Tissues were fixed in 70% ethanol containing 0.02% diethyl pyrocarbonate (DEPC), processed routinely, embedded in paraffin, and cut into 10 µm-thick sections for hematoxylin and eosin (H&E) and Alcian blue staining, and into 5 µm-thick sections for immunofluorescence (IF) and immunohistochemistry (IHC). The same fixation condition was applied to all groups for comparative histological and immunostaining analyses.

### 4.10. Hematoxylin and Eosin Staining

Paraffin-embedded liver sections (10 µm-thick) were deparaffinized twice in xylene for 10 min and rehydrated using graded ethanol. The sections were stained with hematoxylin (Fisher Scientific, Waltham, MA, USA) for 3 min, rinsed in distilled water, and counterstained with eosin (Fisher Scientific) for 30 s. The sections were then dehydrated, cleared, and mounted using Permount (Thermo Fisher Scientific, Waltham, MA, USA).

### 4.11. Alcian Blue Staining

Paraffin-embedded liver sections (10 µm-thick) were deparaffinized, rehydrated, and stained with Alcian blue to assess acidic mucopolysaccharide-associated signals. After staining, the sections were washed with PBS, mounted using Permount, and analyzed under a light microscope.

### 4.12. Immunofluorescence Staining

For cell IF, the treated cells were washed with PBS, fixed in 4% formaldehyde for 30 min at room temperature, permeabilized with 0.2% Triton X-100 in Tris-buffered saline (TBS), and blocked before antibody incubation. The cells were incubated overnight at 4 °C with anti-TNF-α antibody (ab6671; Abcam, Cambridge, UK) diluted 1:300 in blocking buffer. Cellular actin and nuclei were visualized using a staining solution containing phalloidin and DAPI, each diluted at 1 µL/mL in 1× Tris-buffered saline (TBS).

For tissue IF, paraffin-embedded liver sections (5 µm-thick) were deparaffinized, rehydrated, and incubated with primary antibodies against BCL-2 (sc-492; Santa Cruz Biotechnology, Dallas, TX, USA) and caspase-3 p17 (sc-373730; Santa Cruz Biotechnology) overnight at 4 °C. After washing, the sections were incubated with Alexa Fluor 488-conjugated goat anti-rabbit IgG (ab150077; Abcam) or Alexa Fluor 594-conjugated goat anti-mouse IgG (AB150116; Abcam), followed by nuclear counterstaining with DAPI. Cell images were acquired using an ECLIPSE Ti2-U microscope, and tissue images were acquired using a Ni-U fluorescence microscope (Nikon, Tokyo, Japan). Image acquisition and channel merging were performed using NIS-Elements software (BR 5.42.01, 64 bit, Nikon), and signal quantification was performed using Fiji ImageJ software (version 2.16.0/1.54p, National Institutes of Health, Bethesda, MD, USA).

### 4.13. Immunohistochemistry

Paraffin-embedded liver sections (5 µm-thick) were deparaffinized, rehydrated, and subjected to antigen retrieval in 10 mM sodium citrate buffer. Endogenous peroxidase activity was quenched using 3% hydrogen peroxide for 5 min. The sections were then blocked for 1 h and incubated overnight at 4 °C with primary antibodies against IGF-IRα (sc-712; Santa Cruz Biotechnology), mTOR (7C10; Cell Signaling Technology, Danvers, MA, USA), and PCNA (sc-7907; Santa Cruz Biotechnology), all diluted 1:300 in blocking buffer. After washing, the sections were incubated with biotinylated secondary antibodies, followed by incubation with an avidin-biotin complex reagent and a metal-enhanced DAB substrate (Invitrogen, Thermo Fisher Scientific, Waltham, MA, USA). The sections were counterstained with hematoxylin, mounted using Permount, and observed under a light microscope.

### 4.14. Western Blot Analysis

Proteins were extracted from the cells or liver tissues using Pro-PREP protein extraction solution (17081; iNtRON Biotechnology, Seongnam, Republic of Korea). Equal amounts of protein (50 µg) were separated by SDS-PAGE and transferred onto polyvinylidene difluoride membranes. The membranes were blocked with 3% bovine serum albumin and incubated overnight at 4 °C with the following primary antibodies: β-actin (sc-47778; Santa Cruz Biotechnology), caspase-3 p17 (sc-373730; Santa Cruz Biotechnology), BCL-2 (sc-492; Santa Cruz Biotechnology), TNF-R1 (sc-31349; Santa Cruz Biotechnology), BCL-xL (54H6; Cell Signaling Technology), TIMP-2 (sc-9905; Santa Cruz Biotechnology), TIMP-3 (sc-6836; Santa Cruz Biotechnology), IGF-IRα (sc-712; Santa Cruz Biotechnology), caspase-9 (C9; Cell Signaling Technology), IL-2 (sc-133118; Santa Cruz Biotechnology), PI3-kinase C2α (sc-365290; Santa Cruz Biotechnology), Akt1 (sc-5298; Santa Cruz Biotechnology), and p53 (1C12; Cell Signaling Technology). After washing, the membranes were incubated with appropriate secondary antibodies for 2 h at room temperature. Signals were detected using an electrochemiluminescence substrate (WP20005; Invitrogen, Thermo Fisher Scientific) and visualized using a Kodak Image Station 4000MM (Eastman Kodak Company, Rochester, NY, USA). Band intensities were quantified using ImageJ and normalized to the corresponding β-actin signal.

### 4.15. Antibody-Based Colorimetric Assay

Relative antibody-reactive signals in liver tissue lysates were examined using an antibody-based colorimetric plate assay. Tissue lysates were prepared as described for Western blot analysis. Target-specific primary antibodies were coated onto 96-well plates at 4 °C for 24 h. After washing, the plates were blocked with 1% skim milk and incubated with tissue lysates, followed by horseradish peroxidase-conjugated secondary antibodies. Color was developed using substrate solution (R&D Systems, Minneapolis, MN, USA), and the reaction was stopped with 1 M H_2_SO_4_. Absorbance was measured at 450 nm using an Epoch Microplate Spectrophotometer (Agilent BioTek, Agilent Technologies). The assay did not include purified protein standards; therefore, the results were expressed as relative absorbance signals and used only for within-assay group comparisons. The primary antibodies used in this assay were TNF-R1 (sc-31349; Santa Cruz Biotechnology), IL-6 (ab9324; Abcam), caspase-3 p17 (sc-373730; Santa Cruz Biotechnology), BCL-2 (sc-492; Santa Cruz Biotechnology), IGF-1 (ab223567; Abcam), PI3-kinase C2α (sc-365290; Santa Cruz Biotechnology), and Akt1 (sc-5298; Santa Cruz Biotechnology).

### 4.16. Gelatin Zymography

Gelatinase activity in liver tissue lysates was evaluated using gelatin zymography. Protein samples were mixed with FOZ buffer, and 25 µg of protein was loaded onto gelatin-containing SDS-PAGE gels. Electrophoresis was performed at 130 V for 90 min. The gels were washed in renaturation buffer containing 2.5% Triton X-100 and incubated in zymography reaction buffer containing Tris-HCl, NaCl, CaCl_2_, ZnCl_2_, Triton X-100, and NaN_3_ at 37 °C for 18 h. Gels were stained with Coomassie Brilliant Blue R-250 (Biosesang, Yongin-si, Republic of Korea) and destained until clear proteolytic bands were observed. Gelatinolytic band intensities were quantified using ImageJ after background subtraction.

### 4.17. Image Analysis

IF and IHC images, as well as Western blot and zymography bands, were analyzed using ImageJ software (National Institutes of Health, Bethesda, MD, USA). For image-based assays, regions of interest were defined uniformly across groups, and positive signals were measured after background subtraction using the same threshold settings within each assay. For Western blotting, target band intensities were normalized to the corresponding β-actin signal. For gelatin zymography, gelatinolytic band intensities were measured after background subtraction and expressed relative to the NC group.

### 4.18. Statistical Analysis

Cell-based experiments were performed in at least three independent replicates, with each conducted in technical triplicate. Animal/tissue-based quantitative comparisons, when generated, were based on the experimental animal groups described above, with each mouse considered a biological unit. Data are presented as mean ± standard error of the mean (SEM). Statistical analyses were performed using IBM SPSS Statistics version 21 (IBM, Armonk, NY, USA). Differences among groups were analyzed by one-way analysis of variance followed by Tukey’s post hoc test when the data met the assumptions of normality and homogeneity of variance. When these assumptions were not met, the Kruskal–Wallis test followed by Bonferroni-corrected pairwise comparisons was used. A value of *p* < 0.05 was considered statistically significant.

## 5. Conclusions

The present study showed that TLE exposure induced injury-associated responses in hepatocytes and histological alterations in mouse liver tissue. ASE post-treatment changed selected apoptosis-, inflammation-, ECM-, and IGF-related markers after TLE exposure, but the response was not consistently protective across all experimental conditions. ASE alone also increased apoptosis-associated signals in hepatocytes at the tested concentration. Because serum liver injury markers were not measured, ASE was not chemically standardized, no positive-control hepatoprotective compound was included, and collagen/fibrosis-related endpoints were not assessed, these findings should be interpreted as histological and tissue-level molecular changes within the limits of the present experimental design. Further studies using standardized ASE preparations, ASE dose–response testing, liver enzyme analysis, positive-control treatment, fibrosis marker assessment, and direct pathway validation are required to determine whether ASE has reproducible effects on liver injury-related outcomes.

## Data Availability

The original contributions presented in this study are included in the article. Further inquiries can be directed to the corresponding author.
